# The New Old (and Old New) Medical Model: Four Decades Navigating the Biomedical and Psychosocial Understandings of Health and Illness

**DOI:** 10.3390/healthcare5040088

**Published:** 2017-11-18

**Authors:** Albert Farre, Tim Rapley

**Affiliations:** 1 Institute of Applied Health Research, University of Birmingham, Birmingham B15 2TT, UK; 2 Department of Social Work, Education and Community Wellbeing, Northumbria University, Newcastle upon Tyne NE7 7XA, UK; tim.rapley@northumbria.ac.uk

**Keywords:** medical philosophy, psychosocial aspects, health care delivery, attitude of health personnel, doctor-patient relations, medical sociology

## Abstract

The importance of how disease and illness are conceptualised lies in the fact that such definition is paramount to understand the boundaries and scope of responsibility associated with medical work. In this paper, we aim to provide an overview of the interplay of these understandings in shaping the nature of medical work, philosophically, and in practice. We first discuss the emergence of the biopsychosocial model as an attempt to both challenge and broaden the traditional biomedical model. Then, we outline the main criticisms associated with the biopsychosocial model and note a range of contributions addressing the shortcomings of the model as initially formulated. Despite recurrent criticisms and uneven uptake, the biopsychosocial model has gone on to influence core aspects of medical practice, education, and research across many areas of medicine. One of these areas is adolescent medicine, which provides a particularly good exemplar to examine the contemporary challenges associated with the practical application of the biopsychosocial model. We conclude that a more optimal use of existing bodies of evidence, bringing together evidence-based methodological advances of the biopsychosocial model and existing evidence on the psychosocial needs associated with specific conditions/populations, can help to bridge the gap between philosophy and practice.

## 1. Introduction

Medical work can be understood as a set of practices (such as listening, asking questions, diagnosing, or recommending treatments) undertaken by doctors to help those who seek medical attention. At the core of such medical work is the definition of ‘disease’ and ‘illness’, two terms rooted in different understandings of people’s ‘ill health’.

Whilst the term ‘disease’ would typically have a strictly physiologically based definition, the term ‘illness’ would typically be defined in terms of the human experience of ‘ill health’, encompassing both an objective and a subjective reality in its definition. As Idler [1] describes it:

*‘it is possible for an individual to have a disease, yet be unaware of it and act accordingly; it is also possible for people to feel and/or act sick without showing evidence of any objectively verifiable disease. In the former instance there is no illness, though there may be disease. In the latter case there is certainly illness’*.

(p. 723)

Thus, the importance of how the object of medical work is conceptualised (i.e., in terms of disease or illness) lies in the fact that such a definition is paramount to understand the boundaries and scope of responsibility associated with such work [2,3,4].

Hence, a ‘narrow definition’ of the object of medical work in terms of disease—as strictly concerned with organic malfunction—will translate into a medicine exclusively concerned with the physical aspects of illness. In this way, disease is a biomedical concept. On the other hand, a ‘broad definition’ of the object of medical work in terms of illness—as concerned with the life world of the patient—will translate into a medicine that directs clinical attention to all domains of human life, comfortable in the idea that the boundaries between health and illness, between well(ness) and sick(ness), are diffused by cultural, social, and psychological considerations [3].

The aim of this paper is to provide an overview of the interplay of these conceptual approaches in shaping the nature of medical work, philosophically and in practice. We first discuss the rise of the biopsychosocial model in medicine as an attempt to both challenge and broaden the traditional biomedical model. We then synthesise the key controversies and criticisms associated with it, and illustrate the current relevance and engagement with the model before outlining some concluding remarks in light of the subject matter of this special issue, adolescent health and medicine.

## 2. Questioning the Biomedical Approach: The Rise of the Biopsychosocial Model in Medicine

Across a set of papers published between 1960 and 1980 [2,3,5,6,7], George Engel articulated an influential questioning of the historically dominant model of medicine, the biomedical model. He outlined the limitations of such approach and called for the need of a new medical model—which Engel himself characterised as the ‘biopsychosocial model’ [3,8].

Following Engel’s critique, the traditional biomedical approach, which ‘assumes disease to be fully accounted for by deviations from the norm of measurable biological (somatic) variables’ [3], leaves no room within its framework for the social, psychological, and behavioural dimensions of illness. Engel argued that this led to a fundamental paradox that ‘some people with positive laboratory findings are told that they are in need of treatment when in fact they are feeling quite well, while others feeling sick are assured that they are well’ [3] and went on to establish that:

*‘the existing biomedical model does not suffice. To provide a basis for understanding the determinants of disease and arriving at rational treatments and patterns of health care, a medical model must also take into account the patient, the social context in which he [sic] lives, and the complementary system devised by society to deal with the disruptive effects of illness, that is, the physician role and the health care system. This requires a biopsychosocial model’*.

(p. 132)

In other words, Engel proposed to broaden the biomedical approach to include the psychosocial without sacrificing the advantages of the biomedical approach [7] so that ‘patients would continue to be cared for from a disease standpoint but, additionally, psychological and social information would be given equal standing in the care process’ [9].

In doing so, a health professional should be able to evaluate all the factors contributing to illness, whilst recognising some factors as more important than others, and some even as a necessary condition for (as opposed to the cause of) illness, rather than giving primacy to biological factors alone [2]. With that in mind, Engel noted that basic professional knowledge and skills should span the social, psychological, and biological given that medical decisions and actions on the patient’s behalf involve all three domains [3].

Engel’s proposal was theoretically informed by the general system theory [10,11], which is based on the idea that all entities (systems), from the smallest discernible system in physics to the largest system in the cosmos, are structurally and functionally interconnected from level to level with continuous feedback loops [9]. Engel argued that such a conceptual approach was well-suited for his proposed biopsychosocial concept and had the potential to mitigate the holism–reductionist dichotomy as well as improve communication across scientific disciplines [3].

**Figure 1:**
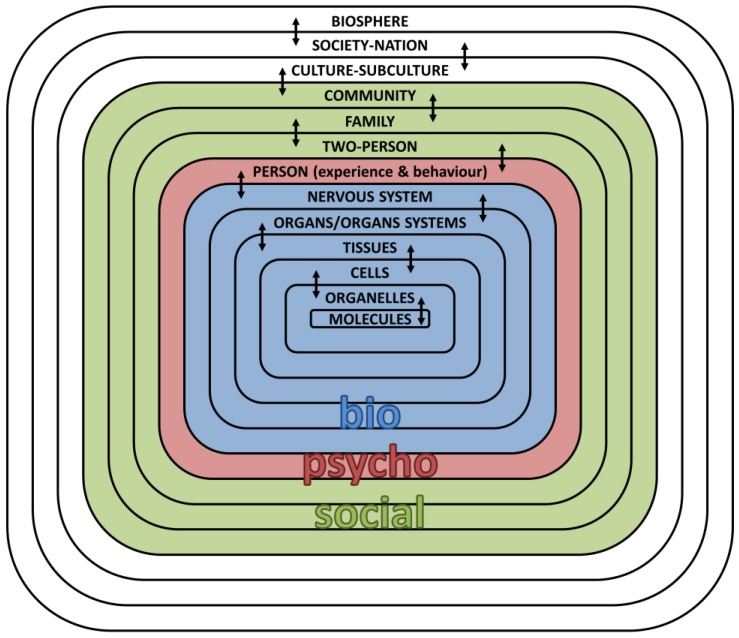
Schematic representation of the hierarchy and continuum of natural systems as applicable to Engel’s definition of the biopsychosocial model—adapted from ‘The clinical application of the biopsychosocial model’ [7].

By applying such reasoning to medicine, Engel defined the biopsychosocial model as encompassing information from the levels below and above the human being as experienced by each person—that is, the health professional seeks to integrate data from the human/psychological level with data from the biological level (below) and data from the social level (above) to construct the biopsychosocial description of each patient (Figure 1).

According to this framework, it must be acknowledged that each level in the hierarchy operates according to a unique system (e.g., tissues and organs at biological level; perception and experience at psychological level; attribution of meaning at social level); however, it is the integration of these systems that is critical in terms of understanding the patient’s biopsychosocial story. Thus, patient-health professional communication becomes a fundamental stepping stone to integrating the various levels and understanding illness and help-seeking behaviour [9,12].

Given that Engel’s proposal was theoretically informed he also argued that, with subsequent empirical support, not only the model had the potential to translate into more humanistic care, but also to make medicine more scientific [9]. However, whilst it is widely accepted that the biopsychosocial model has the potential to lead to more humanistic and patient-centred care, there have been a range of recurring criticisms and controversies associated to its rise, including its amenability to scientific inquiry, as we go on to explore in the following section.

## 3. The Biopsychosocial Model in Medicine: Key Controversies and Criticisms

Following Smith et al. [9], the major criticisms of the biopsychosocial model fall into three broad, overlapping categories:

1. *The model was too vaguely defined and therefore not testable.* A number of authors have suggested that a core limitation of the model, as originally formulated by Engel, was the conceptual underdevelopment [13,14] and lack of operationalisation [15,16], which involved the compromise that the model was not ready to be empirically tested. Some authors such as McLaren [17] even suggested that the model cannot be referred to as a ‘model’, given that it does not conform to the notion of ‘model’ understood as a formal working, representation of an idea or theory that can be empirically tested and holds some predictive and/or explanatory power.
2. *The model’s scope was too generic and cannot be efficiently put in practice.* Other authors have emphasised that the formulation of the biopsychosocial model is so generic in its scope that provides little guidance to health professionals [16] and raises the problem of how to selectively apply the model without any accompanying criteria to locate and specify relevant patient information [18,19]. This can result in an overwhelming scope of loosely related biopsychosocial data that renders the model too time-consuming and inefficient to be applicable for individual patients in practice [20], leaving some to wonder ‘whether there can be a point of diminishing returns in fighting reductionism with inclusionism’ [21].
3. *The model did not include a method to identify relevant biopsychosocial data.* Some authors noted that the model focuses on the need to elicit biopsychosocial information without providing any methodological guidance to assist this process [17]. Within this, critics have also pointed out that the model does not indicate what level of analysis (biological, psychological, or social) to prioritise or when [19], and, since it is often not known which factor might be the ultimate responsible for a given condition, all levels of analysis routinely co-exist and clinicians are left to choose the level that seems to work best [22], without a shared rationale as to why a given clinician heads in one direction or the other [23].

Alongside these, various authors, including most critics, have addressed what they saw as shortcomings of the biopsychosocial model as initially formulated and a number of ‘solutions’ have been proposed over the years.

For example, Schwartz & Wiggins [18] proposed a phenomenological model, addressing the ‘weaknesses’ of the biopsychosocial model by focusing on the fundamental ‘necessity of understanding’ (that is, the doctor’s need to ‘understand patients’) which, they argued, emphasised ‘the relevance of both the human and natural sciences to medical science’.

Later on, Foss & Rothenberg [15] suggested that Engel’s biopsychosocial model (and its limitations/critiques) had to be read as a transition between the various compromises associated with the biomedical model and a more comprehensive model, which they went on to formulate and called the ‘infomedical model’ [15].

However, the idea of a biopsychosocial approach to medical work seemed to have struck a particularly resonant chord, and neither the criticisms nor the calls for new, improved models overshadowed the appeal of Engel’s proposal. Thus, regardless of how ‘flawed’ it was as a model, the idea of a biopsychosocial approach that would improve but not alienate the traditional biomedical approach, resonated with various sectors of the medical profession that wanted to see medical practice informed by a more encompassing understanding of health and illness, more in tune with the actual experiences of those who seek care [24]. Despite the criticisms, the biopsychosocial model went on to influence core aspects of medical practice, education, and research.

## 4. The Relevance of the Biopsychosocial Model in Current Practice, Research, and Policy

Despite the recurrent criticisms since the biopsychosocial model found its way into the mainstream debates in the medical profession and an arguably historically uneven uptake of psychosocial understandings of health and illness in practice and research [25], the broad principles of the biopsychosocial model have increasingly been echoed in guidance and policy documents. Over the last four decades, the very concept of health has transformed from the traditional biomedical definition as ‘absence of disease’ to a more encompassing understanding rooted in a more psychosocial understanding of health and illness.

Likewise, the core problems and limitations associated with Engel’s initial formulation of the model have increasingly been addressed in the context of ‘solutions’ aimed at appending or complementing, rather than replacing, the biopsychosocial model [26]. For example, Herman [20] suggested that the biopsychosocial model raised the need for a transitional, more pragmatic model (the split model) which ‘relegates the psychosocial to the position of being just another tool in the doctor’s bag’ but makes the task of thinking ‘biopsychosocially’ attainable for health professionals in practice. Kontos [21] has argued that the complexity of contemporary medicine is not suited for a single model. Other authors have formulated a range of contributions in terms of ‘further developments’ [24], including teachable ‘habits of mind’ with the potential to enable a realistic connection between the biopsychosocial vision and the clinical reality [27]; ‘addendums’ [19] and ‘ways to realise’ [28] the biopsychosocial model; ‘strategies’ for bridging and better integrating its three levels of analysis in practice and research [29,30]; more recently, Smith et al. [9] have proposed a more encompassing ‘solution’ by addressing the following question: Exactly *how* do health professionals efficiently identify essential biopsychosocial data when caring for an *individual* patient at a given point in time?

This proposal was in essence a methodological response to the three core criticisms noted above, arguing that the availability of ‘a repeatable method that consistently identifies only the relevant biological, psychological, and social information needed to define the BPS [biopsychosocial] model at each visit’ would make the model scientific and enable further improvements in the clinical, educational, and research arenas. In Smith et al.’s [9] view, this method was to focus on the most important source of biopsychosocial data in each clinical encounter, i.e., the medical interview. Thus they suggested that the biopsychical model could be operationalised by integrating two evidence-based, behaviourally defined patient-centred interviewing methods: the ‘integrated patient-centred and doctor-centred interview model’ and the ‘four habits interviewing model’. These are known to ‘produce highly relevant disease, personal/social, and emotional information’ rather than all biopsychosocial data [9] and have been associated with effective and efficient learning [31,32] and positive health outcomes [33,34] via randomised controlled trials. By providing the biopsychosocial model with a method transformed the general model initially formulated by Engel into a specific model for each clinical encounter, translating the model into an evidence-based, consistently defined, intervention with the potential to address its core shortcomings.

Similarly, debates and changes in the medical education arena also reflect this trend [35] with calls for reforms in medical education to transform primarily biomedical-focused curricula into more encompassing programmes that also incorporate learning from behavioural and social sciences, enabling medical educators to appropriately respond to the need for increased psychosocial competence of future medical professionals [36,37,38].

Accounting for a similar trend, official bodies such as the National Institute for Health and Care Excellence in the United Kingdom or the Institute of Medicine in the United States have acknowledged the need to address the psychosocial dimension of patients’ health concerns, through the implementation of more and improved patient-centred practices, as a way to improve the quality of health care [39,40,41]. Although, as Wade and Halligan noted [42], the biopsychosocial model has had little influence on the larger scale organisation, funding, and commissioning of health care services.

Thus, there is now a greater need to apply the biopsychological model to healthcare management to respond to its growing uptake in practice and research [42], where there have been increasing reports of the application and use of the biopsychosocial model—with recently published examples ranging from cardiology [43] to oncology [44], general paediatrics and internal medicine [45], orthopaedics [46], and obstetrics and gynaecology [47], among many other areas where attempts have been made to implement and evaluate the biopsychosocial model [48,49].

## 5. A Question of Method and Discipline: The Future of the Biopsychosocial Model through the Lens of Adolescence Medicine

Whilst further work is still needed to address some limitations and emerging problems associated with the practical application of the biopsychosocial model both in clinical practice and research, sustained philosophical and political traction is translating in increasing examples from across all areas of medicine. These are already paving the way to further develop the biopsychosocial model in light of concerns previously raised.

One of these areas is adolescent health and medicine [45,50,51]. The provision of health services for young people provides a particularly good exemplar to examine the contemporary challenges associated with the practical implementation of the biopsychosocial model. Firstly, adolescent health care has historically been geared towards a more biopsychosocial understanding of health and illness: Its core principles are ‘based on a biopsychosocial approach to clinical interactions’ [52], aiming to provide a ‘complete and thorough physical and psychosocial evaluation and treatment in an atmosphere of trust and confidentiality’ [53] to better meet the complex and inherently all-encompassing nature of developmental needs of adolescents and young adults [54]. Secondly, because the age range of young people—that is, 10–24 years [55]—locate adolescent services right at the intersection of paediatric and adult care, this provides a unique opportunity to explore such challenges in the context of the wider organisation of the health care system, particularly for those with chronic conditions who will need to move from child- to adult-focused care [41].

However, bridging the gap between philosophy and practice to better integrate the biomedical and psychosocial facets of health and illness is still challenging, even in areas such as adolescent medicine, where the model advocated for by adolescent health specialists is often far from the experiences of young people using health services [56] and where different approaches to the integration between the biomedical and psychosocial facets of healthcare coexist among clinicians [57].

Nevertheless, a more optimal use of existing bodies of evidence can help to bridge this gap between philosophy and practice. In particular, changes in actual clinical practice could be facilitated by bringing together evidence-based methodological advances of the biopsychosocial model and existing evidence on the psychosocial needs associated with specific conditions/populations, thus helping to further tailor and aid the practical implementation of the model and proposed method through specialty/condition/population related guidance for clinicians. For example, in terms of adolescent medicine:

- On the one hand, the pivotal advance introduced by Smith et al. [9] linking the biopsychosocial model to an evidence-based patient-centered interview method, addresses the three major concerns with the biopsychosocial model and enables the operationalisation of the biopsychosocial model for its use in each consultation.
- On the other hand, there is a growing but well-established body of evidence on the specific health and psychosocial needs of adolescents that should be addressed in each consultation. These are detailed in the reviewed Home, Education/Employment, Eating, Activities, Drugs, Sexuality, Suicidal ideation and Safety (HEEADSSS) tool [58,59,60], a psychosocial interview tool which has been described as the gold standard in obtaining a developmentally appropriate psychosocial history from young people [61].

Building on the methodological advances by Smith et al. [9], the practical implementation of the biopsychosocial model could be facilitated by further tailoring the description of the proposed patient-centered interview method, drawing on evidence-based recommendations and guidance on the psychosocial needs for a given specialty/condition/population.

In addition, it is equally important to consider that policy frameworks and clinical guidelines also have the potential to enable further advances in the practical implementation of the biopsychosocial model in a number of ways. For example, by building psychosocial aspects of health and illness into clinical governance and the judgment of the quality of care, or by redefining and devising new roles to further ingrain multi-disciplinary and trans-disciplinary work in routine clinical practice. Equally, establishing an applied health research agenda, responsive to the multidisciplinary and complex nature of the psychosocial understandings of health and illness, can inform the further development (both conceptual and methodological) and further refining and reshaping of an integrated model that successfully integrates the biomedical and the psychosocial understandings of health and illness in medical work.
